# Impact of variation in climatic parameters on hydropower generation: A case of hydropower project in Nepal

**DOI:** 10.1016/j.heliyon.2022.e12240

**Published:** 2022-12-14

**Authors:** Raj Singh, Nawraj Bhattarai, Anita Prajapati, Shree Raj Shakya

**Affiliations:** a Pulchowk Campus, Institute of Engineering, Tribhuwan University, Kathmandu, Nepal; b Institute for Advanced Sustainability Studies, Potsdam, Germany

**Keywords:** Hydropower generation, Climate change, Run-off-river (ROR), WEAP model, Climatic parameters, Temperature, Precipitation, Streamflow

## Abstract

Nepal has substantial potential to generate electricity through hydropower projects. Most of the hydropower projects in Nepal are Run-off-River (ROR) types. Significant seasonal variation can be pronounced on its river basins resulting in higher streamflow & higher hydropower generation during the wet/summer season and just reverse scenario in case of the dry/winter season. Thus, ROR-type hydropower in Nepal is more susceptible to Climate Change. This study assesses the impact of variation in climatic parameters on the hydropower generation by implementing WEAP model using the meteorological and hydrological data from 1976 to 2004 under Reference & Climatic Scenarios. The results reveal that the streamflow of Dordi River of Nepal is in increasing trends and can be more pronounced during April, May, June & July of the season under climatic scenarios. The generation of hydropower plant is likely to increase up to 15%, 1%–32% & 1%–51% over the study period under climatic scenario-1, 2 & 3, respectively, as compared to baseline scenario and the increments are observed to be more prominent during April & May of the season which is very crucial finding in current context of Nepal as there is power deficit during the dry season. Therefore, detailed technical and policy level planning can enhance the power generating capability of the future hydropower projects that will be developed in this corridor. This will significantly impacts the national energy planning and implementation.

## 1 Introduction

In the context of Nepal, the majority of the electricity generation is contributed through the hydropower sector. Nepal has tremendous potential to generate electricity through hydropower projects. The country's river basin has a theoretical potential of 83,290 MW, out of which 45,610 MW is technically viable & 42,133 MW is economically feasible [1]. However, it hasn't been able to harness even 5 % of the hydropower potential mentioned above. The present hydropower generation capacity in the country is about 6052 GWhr, and the current peak demand is 1482 MW [2]. On a positive note, the Nepal Government has planned to expand the power generation up to 15,000 MW by 2030, on which a significant contribution will be from the hydropower sector [3].

Most of the hydropower in Nepal is Run-off-River (ROR) type. Thus, significant seasonal variation can be pronounced in the river basins resulting in higher hydropower generation during the wet/summer season while lower generation during the dry/winter season. This seasonal variation causes energy deficits during the dry season. In these conditions, the energy demands are met by importing energy from the neighboring country [2].

Climate Change has been a serious challenge and matter of concern globally, regionally & nationally. Global warming is a key factor of the Climate Change. It is quite evident that the temperatures have been increasing globally and causing serious climate-related risks for human and natural systems. The IPCC Special Report on the impacts of global warming of 1.5 °C stated that it is estimated to cause approximately 1.0 °C of global warming above pre-industrial levels by human activities, with a possible global temperature rise in the range of 0.8 °C–1.2 °C. Global warming will possibly approach 1.5 °C between 2030 and 2052 if it continues to increase at the current trend [4]. Several efforts have been put together globally to respond to the serious threat of Climate Change. Many plans and policies have been formulated and implemented at the national level in the form of Nationally Determined Contribution (NDC) to suppress rising global temperature from the national level*.* The Paris Agreement sets the main goal to limit the global temperature rise this century well below 2 °C above pre-industrial level central and to put efforts to keep temperature rises to 1.5 °C. In addition to this, the agreement intends to improve the nation's capacity to deal with the effects of climate change and align the constant financial flows with low GHG emissions and a climate-resilient pathway.

Climate Change has a greater impact on hydropower. Many studies can be found across the globe assessing the impact of Climate Change on hydropower projects. A study was conducted by Oti el al. [5] in the Densu River Basin. The study showed that the temperature would increase by 8.23 %, and rainfall would be decreased by 17% in that area due to the impact of climate change. An investigation of Olabanju et al. [6] revealed that under RCP 4.5 & 8.5 scenarios, the temperature is likely to increase in the range of 1 °C–4 °C & there will be a decrease in the precipitation in the range of 5%–30% as compared to the baseline scenario.

Liu et al. [7] have researched the impacts of Climate change in the river basins of China. The results of study in the Yiluo River, northern part of China, demonstrated that the mean annual runoff is likely to decrease by 22% & 21% under 1.5 °C & 2 °C temperature increment scenarios, respectively, while it is projected to increase by less than 1% & less than 3% under 1.5 °C & 2 °C scenarios in the Beijing River, southern part of China as compared to the baseline scenario. Similarly, another research in the Upper Yangtze River basin of China was conducted by Chen et al. [8] and observed a slight increase and decrease in the river's annual discharge under 1.5 °C & 2 °C scenarios, respectively.

Nepal has been experiencing visible impact of Climate change over the past few decades. It can be observed that the temperature in Nepal is in increasing trend. The annual maximum temperature has been increasing at the rate of 0.056 °C/year between 1975 & 2014. Likewise, the minimum temperature increases at the rate of 0.02 °C/year mainly pronounced during monsoon season [9]. It is found to have an increasing trend in temperature in the Eastern Koshi river basin & Karnali [10, 11].

Similarly, it can be observed that there is variation in the precipitation due to the impact of Climate Change. The rainfalls are observed to have a decreasing trend during pre-monsoon and post-monsoon, while rainfalls are in increasing trends during monsoon in the Gandaki river basin [12]. The precipitations in various stations of the Karnali river basin are found to show both increasing and decreasing trends. However, the average precipitation is found to have a decreasing trend in most of the stations [11].

The government agency in Nepal has carried out research to assess the patterns of changing Climate in the future periods. It has been projected in the study that average annual precipitation is expected to increase by 8–12% in the long-term and 2–6% in the medium-term period. Likewise, the average annual mean temperature is expected to increase by 0.9–1.1 °C in the medium-term and 1.3–1.8°Cin the long-term [13].

A study has been carried out in the Marsyandi River, Lamjung district of Nepal, regarding the variation of Climatic parameters -temperature & precipitation and projections in future periods in a different scenario. The investigation has revealed that the temperature is likely to increase by 0.47 °C from maximum temperature and 0.84 °C from minimum temperature, 0.96 °C from maximum temperature & 1.33 °C from minimum temperature & 1.18 °C from maximum temperature & 1.49 °C from minimum temperature by 2030s, 2060s & 2090s respectively and precipitation by 6%, 12% & 17% by 2030s, 2060s & 2090s respectively with respect to the value of temperature and precipitation recorded at Khudi Bazar Station, Lamjung under baseline scenario [14].

It can be observed from the above studies and research that the climatic pattern – temperature and precipitation has been dynamically changing in most parts of Nepal. Therefore, the hydropower projects in Nepal are susceptible to Climate Change. A study has carried out in the Gandaki river basin of Nepal and observed that the variations in climatic parameters had impacted the generation of the Trishuli Hydropower Project located in the basin [15]. Likewise, a study was carried out by Sahukhal & Bajracharya [16] at the Kaligandaki gorge HPP, Myagdi district of Nepal to assess the impact on the hydropower plant due to climatic parameter variation implementing LEAP & WEAP software. The study showed there is variation in precipitation patterns in the vicinity of the project area with no any change in the temperature trend. However, the discharge of the Kaligandaki river is found to have a decreasing trend. The investigation in the Kaligandaki river revealed that there is a decrease in full capacity power generation of the Kaligandaki Gorge Hydropower Project. Similarly, the study in the Kaligandaki river basin area revealed that the hydropower potential in that basin has been influenced by the impact of climate change [17].

Currently, on the Dordi Corridor located in the Lamjung district of Nepal, there are several projects that are under construction phases and some are even in the verse of completion, namely - Dordi Khola Hydroelectric Project -27 MW, Dordi-I Hydroelectric Project -10.3 MW, Upper Dordi-A Hydroelectric Project -25 MW & Super Dordi Hydroelectric Project -54MW. Thus, a significant amount of electricity cumulatively 116.3 MW, is going to tap into the national electricity grid when all these projects come in to operation in full swing. However, there has been no any necessary assessment in the Corridor conducted by hydropower developers, project authorities and other stakeholders to consider the potential risks and impacts related to the climate that can be arisen in the future due to the Climate Change. Thus, it is very important to assess the potential impacts on hydropower due to the variation in the Climatic parameters in the corridor and possibly utilize the results for the hydropower development & operation, climate-related risk analysis, and ultimately integrate the results into the national energy planning and implementation.

Therefore, this study focuses on 1) Evaluation of WEAP model performance of Dordi River & 2) Assessment of the impacts on streamflow of Dordi River and power generation of Super Dordi Hydropower Project Kha due to the variation in the climatic parameters.

## 2 Material and methods

### 2.1 Description of Study area

**Figure 1:**
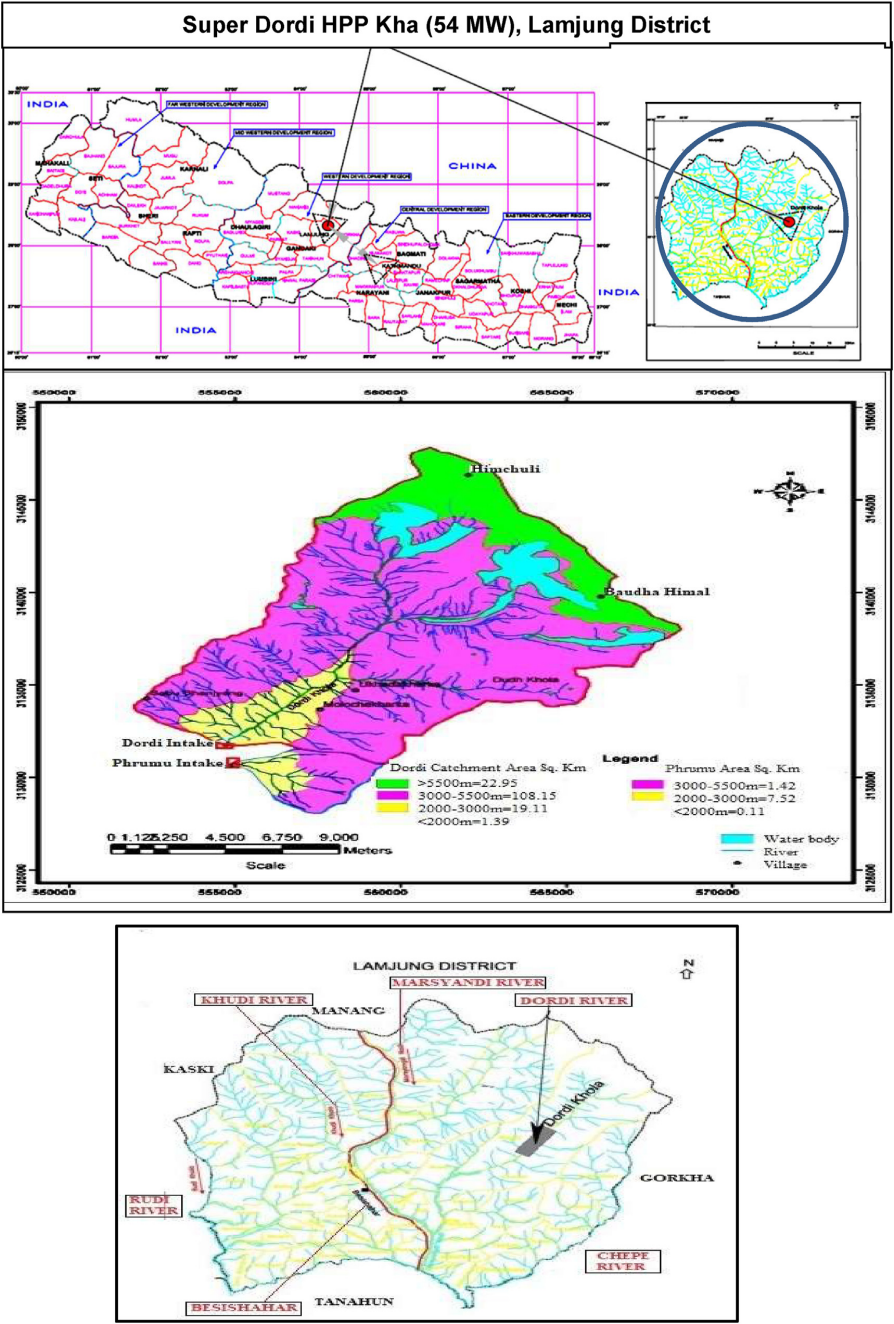
Project location map & catchment of super Dordi HPP Kha [19]. 1.A - Detail of rivers in the Lamjung district.

The Super Dordi Hydropower Project (HPP)-Kha is located in Lamjung District in the Western Development Region of Nepal. It is a Run-off-River type of project being developed by Peoples Hydropower Company Ltd. The Geographical coordinate of the project area lies between Longitudes 84^o^34′15″ E and 84^o^31′00″ E, Latitudes 28^o^18′50″ N and 28^o^16′20″ N as shown in Figure 1 [18, 19].

Dordi River is one of the major tributaries of Marsyandi River in Lamjung district of Nepal, flowing from North to South and westward direction. The river originates from the southern and eastern slope of Himal Chuli (7893m) and the western slope of Baudha Himal (6672m). Dordi River meets the Marsyandi River near Bhoteodar Lamjung, downstream side of Middle Marsyandi Hydropower Project's headwork, and Marsyandi River meets Trishuli River at Mugling. Dordi River comprises several sub-tributaries like Dudh Khola, Phrumu Khola, etc.

The maximum length of Dordi Khola up to intake is about 18 km. The width of Dordi River's catchment above intake varies from 8.3 km-12 km. The total catchment area of the project is 151.6 sq. km of which 22.95 sq. km lies above 5500 masl altitude, 108.15 sq. km lies between 3000-5500 masl, and 19.11 sq. km between 2000-3000 masl & 1.39 sq. km lies below 2000 masl.

The climate of this region is significantly affected by the region's topography. The mean annual rainfall in the Dordi Khola basin is estimated to be 2535mm. The monsoon begins in late June and continues until late September, followed by a dry period. The winter begins in November and continues until February. The climate becomes progressively warmer in February/March and is characterized by hot and dry weather followed by a transitional pre-monsoon period with thundershowers and frequently strong winds until the beginning of the monsoon. The mean annual temperature of the Gandaki basin is 15.4 °C which increases from North to South. In the lower part of the project area, the sub-tropical climate can be experienced during the dry and rainy seasons. However, the upper part of the Dordi River is cold. The area's temperature ranges from 8 °C (in January) to 23 °C (in July). The most mixed dense forest can be found in the Dordi River banks in the vicinity of intake river banks of Dordi near intake are mostly mixed dense forest. There is no settlement at the upstream side of the Dordi intake. A tributary named Prumu River also consists of a dense mixed forest catchment. In the cultivated basin area, the general type of agricultural soil is found which varies from sandy loam to loamy sand and soil depth ranges from 0.15m to 1.83m. The riverside valley on the bottom and the plains tend to be more fertile than the soil on the hill slopes. Barley, wheat, maize, millet, etc., are major crops in this area that are suitable for agriculture. The pasture land also can be found in some of the areas inside the catchment.

### 2.2 Data collection and WEAP model input

The meteorological, hydrological, land use land cover, soil & geographic latitude data are required to model the Dordi River. Twenty-Nine years (1976–2004) of monthly temperature, precipitation, and relative humidity data are obtained from the Department of Hydrology and Meteorology (DHM), Government of Nepal, for the Khudi Bazar Station (Station ID 802) located at Lamjung District of Nepal.

**Figure 2:**
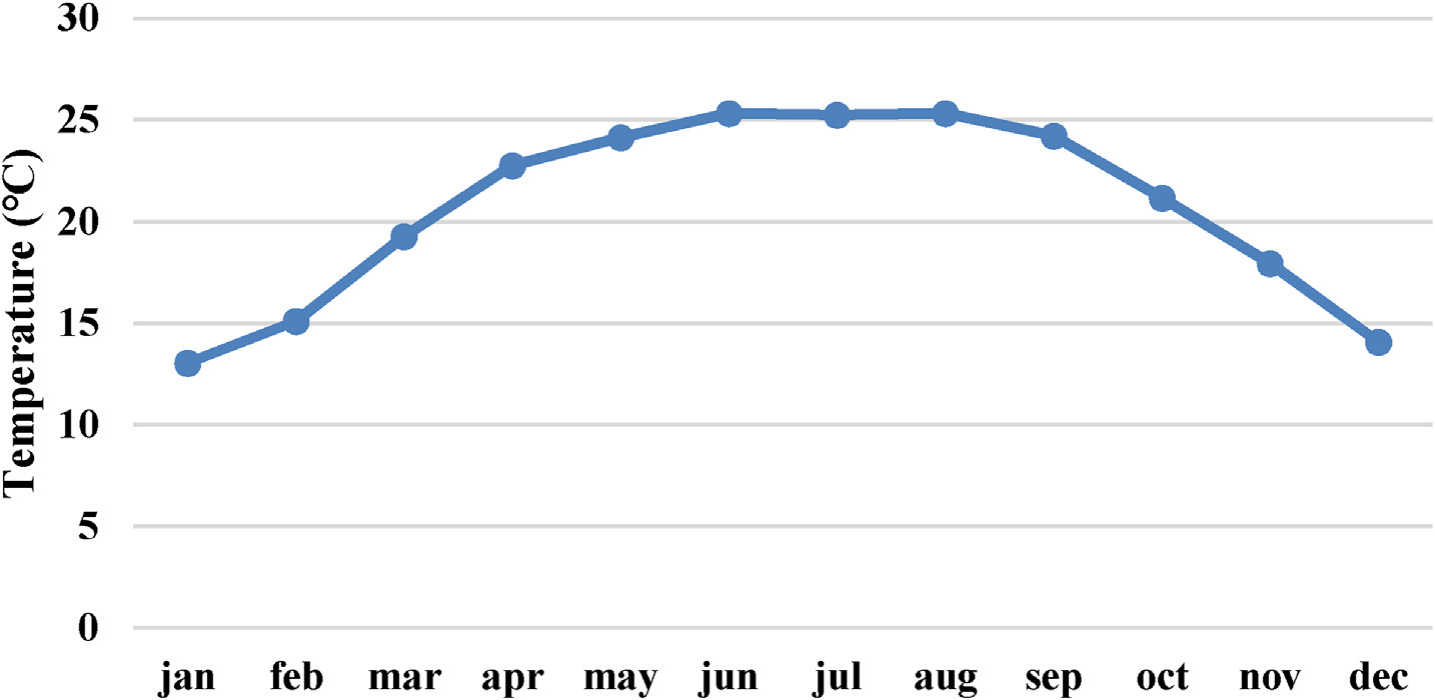
Mean monthly temperature pattern.

**Figure 3:**
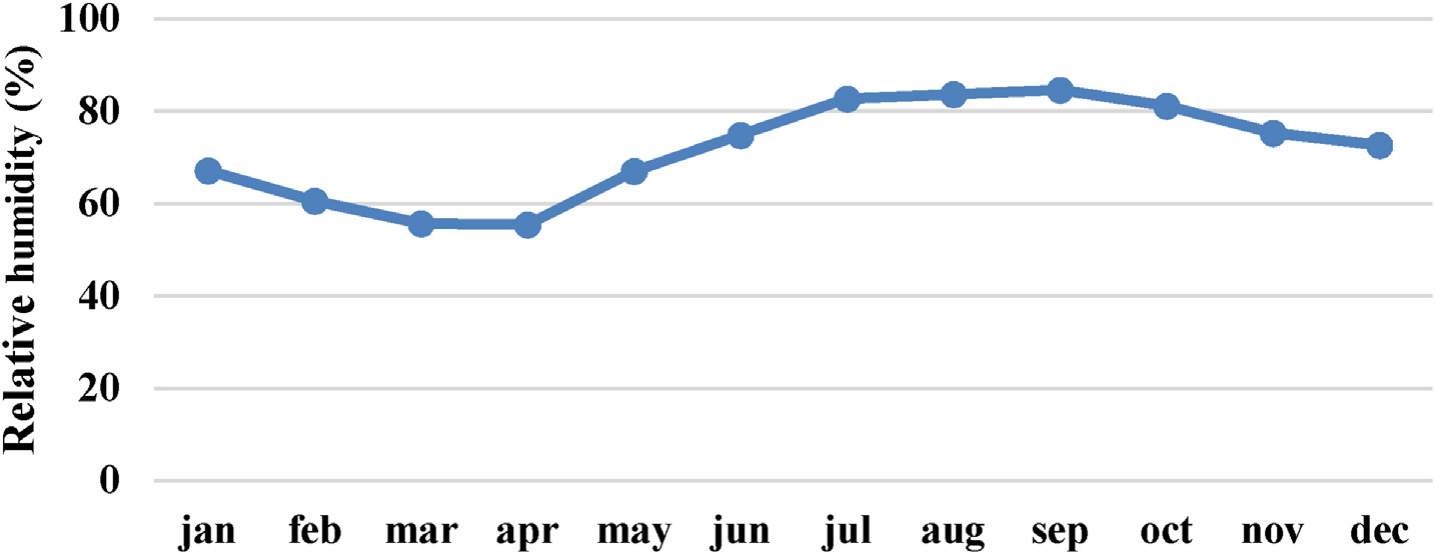
Mean monthly relative humidity.

**Figure 4:**
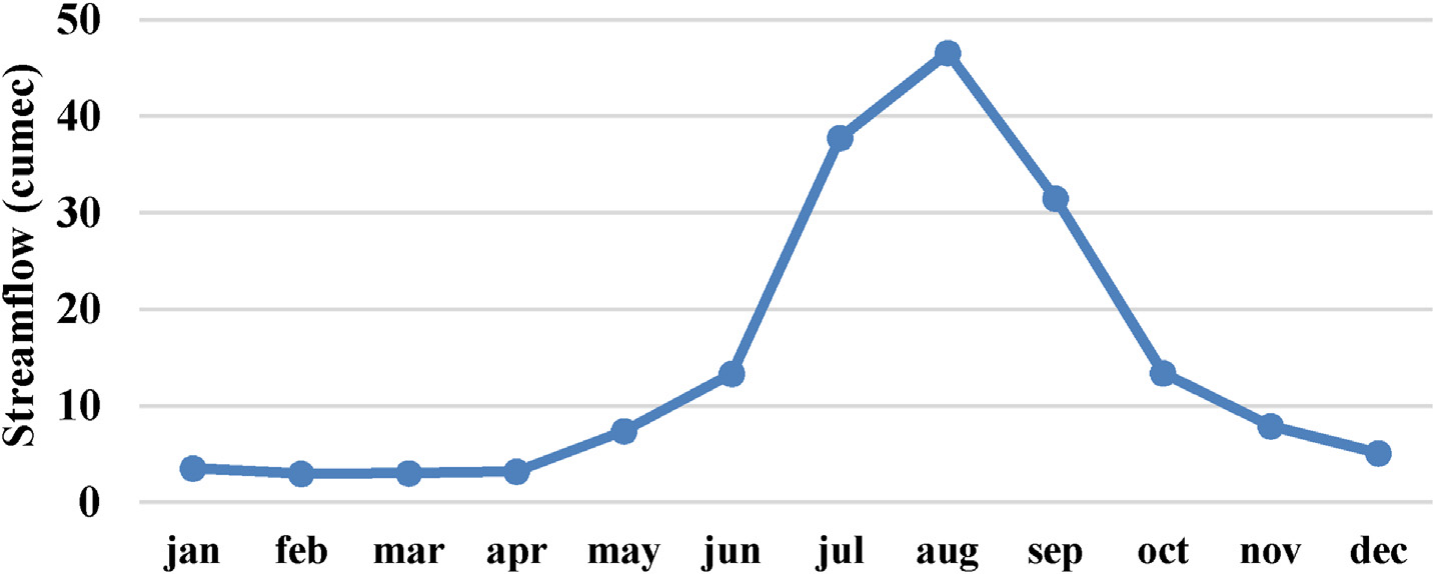
Monthly average precipitation pattern.

**Figure 5:**
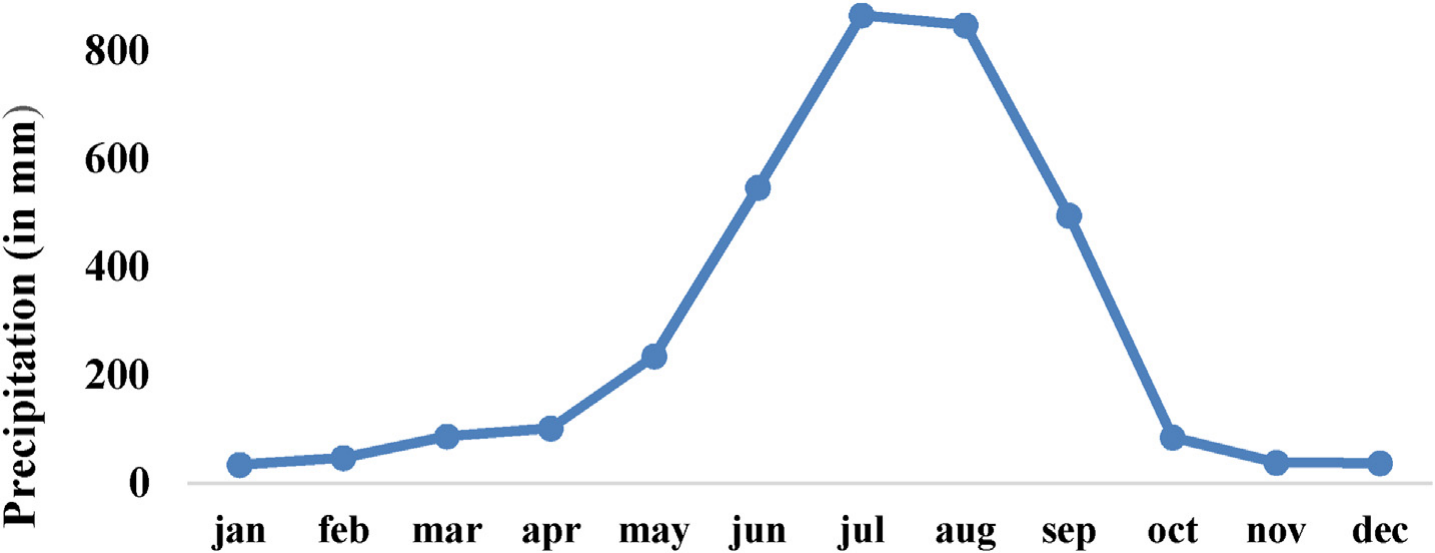
Mean monthly observed streamflow pattern of Dordi River.

The monthly discharge of Dordi River from 1976 – 2004 is obtained from Detail Project Report, DPR [19], 2015, which was recorded by the Peoples Hydropower Co. Ltd & Clean Energy Consultant P. Ltd (developer & design consultant of the Super Dordi Hydropower Project Kha) during the time of project development. And all the missing data are filled by the linear interpolation method. The temperature, discharge, relative humidity & precipitation pattern from the years 1976–2004 is present in Figures 2, 3, 4, and 5.

**Figure 6:**
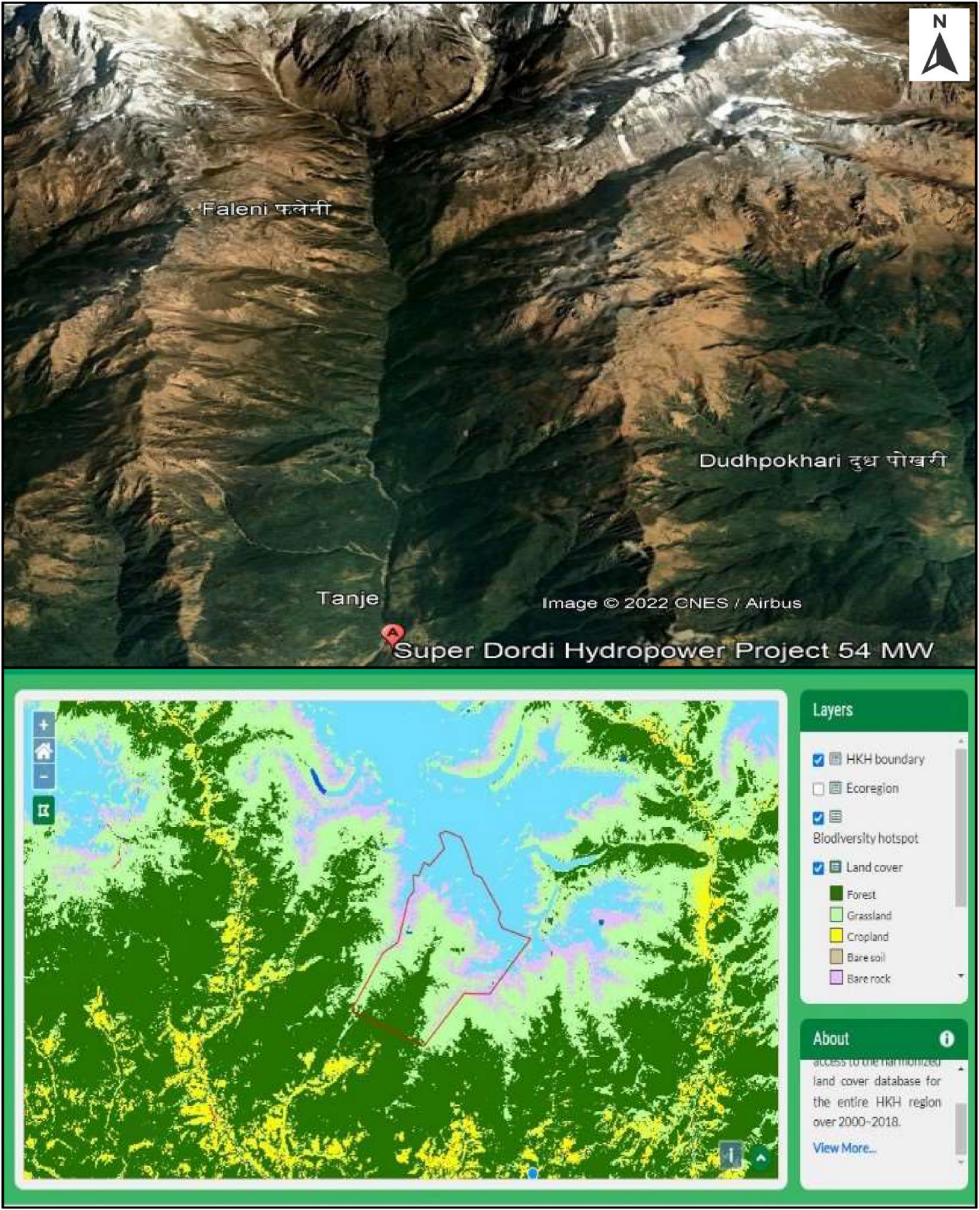
Land use land cover map of Dordi basin (demarcated by red color polyline).

In addition to the Climatic data parameters, the land use and land cover (LULC) are required for the modeling of the Dordi River. The data of the project catchment area: 151.6 Sq. km is fetched from the report produced by Project developer. Further, the land use and land cover map are developed by the tool facilitated by the ICIMOD [20] – land type and their coverage are presented in Figure 6. The Land use pattern of Lamjung District is presented in Table 1.

**Table 1:** Land use pattern of Lamjung district [21].

S.N	Type of Land Use	Lamjung Land Use
1	Agricultural Land	26.45%
2	Forest Land	47.37%
3	Grazing/Pasture Land	13.77%
4	Snow Covered Aarea	2.64%
5	Barren land	6.41%
6	Water covered area	3.30%
7	Other	0.06%

A similar study has carried out by Khadka and Pathak, 2016 in the Marsyandi river basin located in Lamjung district of Nepal [14], located between 27^o^50′42″ N to 28^o^54′11″ N Latitudes and 83^o^47′24″ E to 84^o^48′04″ E Longitudes. It implemented the Second Generation Canadian Earth System (CanESM2) for the Climate Change projection for the future, performed within framework of CMIP5 which contributes to 5^th^ assessment report of IPCC. The CanESM2 climate change scenario has a grid size of 2.8125°. The available data at that resolution wasn't suitable to perform hydrological analysis, therefore, in the study, GCM outputs at a global scale had statistically downscaled to a local scale using a statistical downscaling model (SDSM). Then the output of SDSM was subjected to bias correction using a long term monthly mean to remove any systematic bias. Therefore, CanESM2 is a representative climate model for the climate change impact studies in the Lamjung district, Nepal.

Furthermore, the output of CanESM2 had been downscaled for three RCPs (RCP 2.6, RCP 4.5 & RCP 8.5) to project future temperature and precipitation for the period of 2006–2100 along with NCEP data for 1961 to 2005. The observed data from 1961 to 1995 had been used for the calibration and 1996 to 2005 for the validation.

The Projection for the temperature and precipitation in the future under RCP 4.5 is summarized and presented in Table 2.

**Table 2:** Projected change in temperature & precipitation compared to baseline.

Station		Baseline		RCP4.5			Reference
				2030s	2060s	2090s	
Khudi Bazar	**Temperature**		Projected Change, °C				*Khadka and Pathak, 2019*
	Maximum	26.64 °C		0.47 °C	0.96 °C	1.18 °C	
	Minimum	14.68 °C		0.84 °C	1.33 °C	1.49 °C	
	**Annual Precipitation in Baseline period, mm**	3362mm	% change in Precipitation compared to the Baseline	6%	12%	17%	

Therefore, the present study takes the basis of above climatic results drawn from khudi Bazar Station, Lamjung, Nepal investigated by the khadka and Pathak, implementing CanESM2 dataset with CMIP5 model under RCP 4.5 for the projection of Climatic Parameters (Temperature & Precipitation) for the future periods.

It can be observed from Table 1 that under the RCP 4.5, the temperature is likely to increase by 0.47 °C from maximum temperature and 0.84 °C from minimum temperature (with an average of 0.655 °C), 0.96 °C from maximum temperature & 1.33 °C from maximum temperature (with an average of 1.145 °C) & 1.18 °C from maximum temperature & 1.49 °C from maximum temperature (with an average of 1.335 °C) by 2030s, 2060s & 2090s respectively and precipitation by 6%, 12% & 17% by 2030s, 2060s & 2090s respectively with respect to the value of temperature and precipitation recorded at Khudi Bazar Station, Lamjung under the baseline scenario. Therefore, for projecting the climatic parameters (temperature & precipitation) for future and inputting these projected temperatures and precipitation in WEAP model, three **Climatic Scenarios**: **Climatic Scenario-1, 2 &3** are developed for this study which is presented in Table 3. In the **Climatic Scenario-1,** temperature & precipitation is **increased by 0.5°C & 5 %** respectively, by **1°C & 10 %** in **Climatic Scenario -2** & **1.5°C & 15%** in **Climatic Scenario -3** with respect to the value of mean temperature and precipitation at Reference Scenario.

**Table 3:** Projected change in temperature and precipitation for present study.

Station	Climatic Parameters	Baseline	Projected Change	RCP4.5			Remarks
				2030s	2060s	2090s	
				Climatic Scenario-1	Climatic Scenario-2	Climatic Scenario-3	
Khudi Bazar	Temperature	Reference Scenario	Projected Change, °C	0.5 °C	1 °C	1.5 °C	Climatic Scenario for Present Study
	Precipitation	Reference Scenario	% change in Precipita-tion compared to the Baseline	5%	10%	15%	

In order to assess the uncertainties in thus selected Climatic Scenarios i.e Climatic Scenario-1, 2 & 3, the Scenario analysis has performed for this study using Monte Carlo Simulation Approach and calculated the 95% Confidence Interval in the normal distribution which shows that the temperature and precipitation parameters under Climatic Scenario-1, 2 & 3 lies on the 95% Confidence Interval.

### 2.3 Model Setup

**Figure 7:**
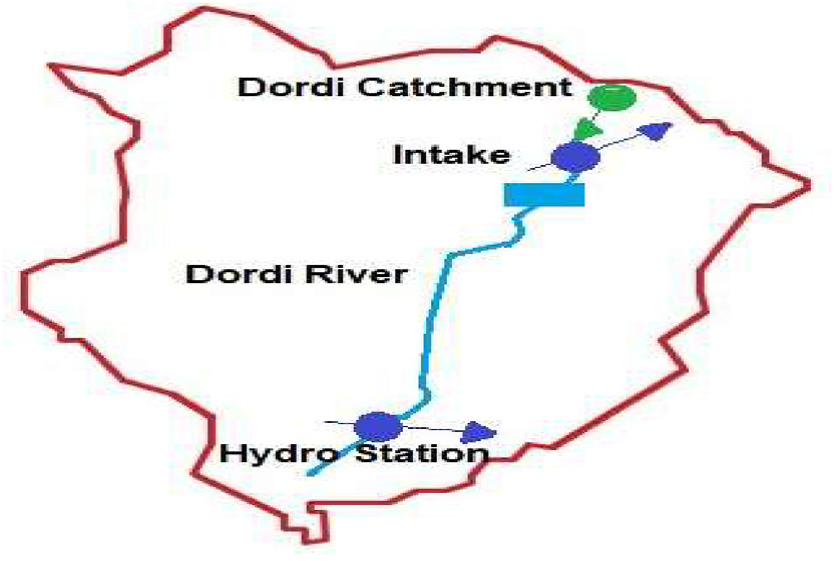
WEAP model set up for the Dordi River.

**Figure 8:**
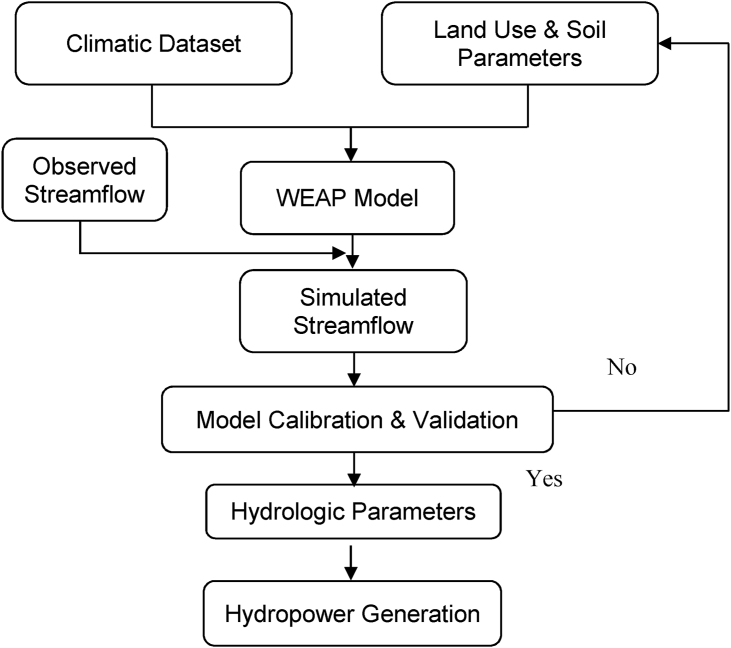
Flow chart for WEAP Hydrologic model.

This study focuses on the development of a hydrological model of the Dordi River via WEAP to assess the hydrological behavior at the Dordi river. The study involves the simulation of the Dordi river through WEAP, the setup of which is shown in Figure 7, and evaluate the impact on the generation of the hydro plant due to the variation in the Climatic parameters under different scenarios – Reference scenario and Climatic Scenario-1, 2 &3 as mentioned above. Figure 8 represents the flow chart for the input, output, and modeling process of the WEAP hydrological model.

The WEAP is a hydrological model developed by the Stockholm Environment Institute (SEI) which is widely used to study the hydrological processes and hydrological cycle [22, 23]. and assess the impact of climate change [24, 25, 26, 27, 28]. The WEAP model includes five methods for modeling the catchment processes – Irrigation Demand Only (Simplified Coefficient Method), Rainfall Runoff (Simplified Coefficient Method), MABIA (FAO 56, dual KC, daily), Rainfall Runoff (Soil Moisture Method) & Plant Growth (daily; CO2, water and temperature stress effects). For this study, the Rainfall Runoff (Soil Moisture Method) is selected among the above other methods to model the Dordi River and assess its hydrological response to the changing climatic parameters due to the availability of the relevant data for the modeling of Dordi River via this method and assess its hydrological response to the changing climatic parameters and it also fits more with the purpose of the present study than other methods. Furthermore, this method accounts for the impact of land use and soil types on these processes.

**Figure 9:**
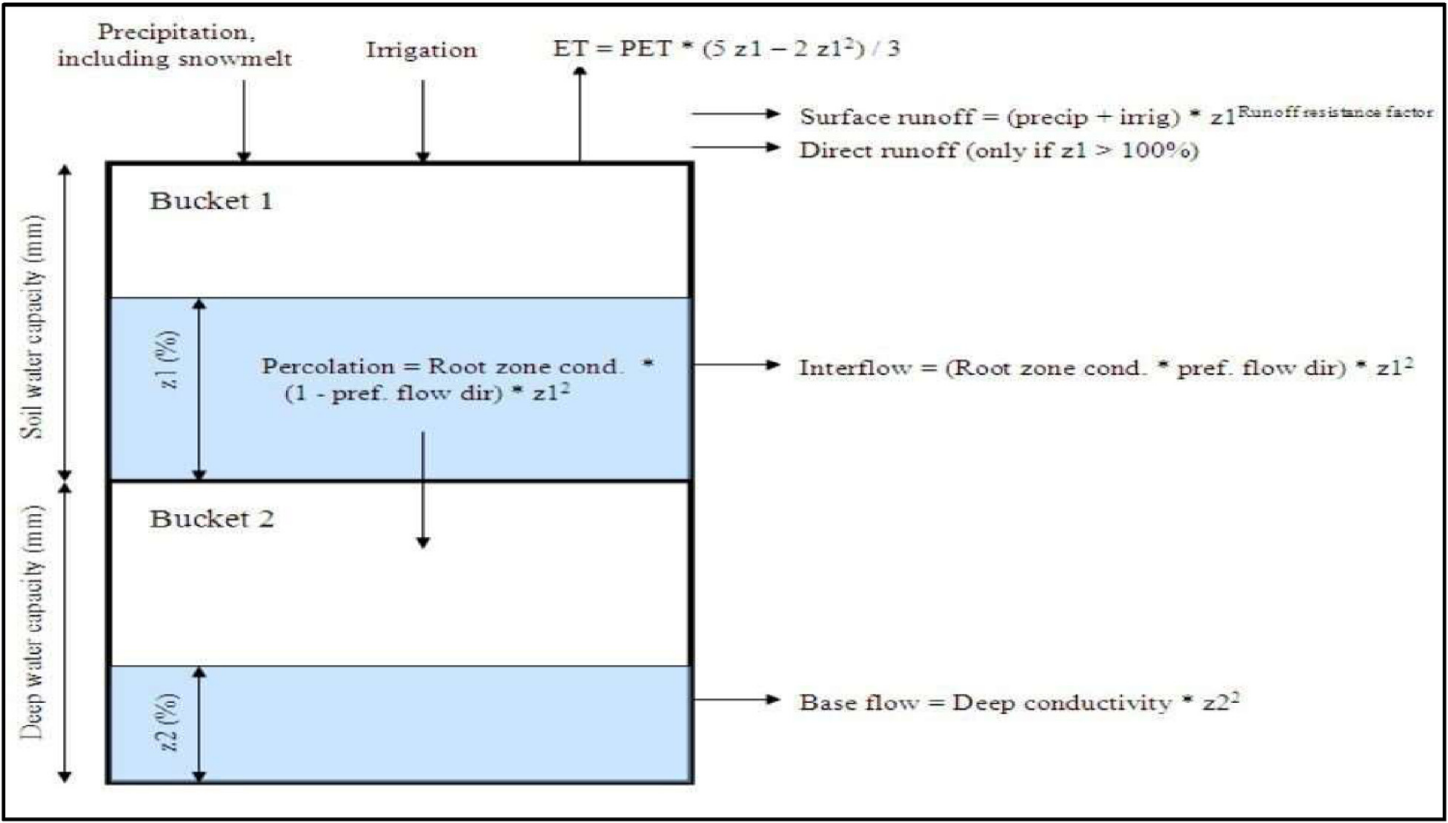
Conceptual diagram and equations incorporated in the Soil Moisture model (Sieber and Purkey, 2015) [31].

In the soil moisture method, the catchment is partitioned into soil layers – the upper soil layer termed shallow water capacity & low soil layer termed as deep-water capacity. This method implements empirical functions that divide the water system into evapotranspiration, surface runoff, sub-surface runoff (i.e., interflow), and deep percolation, as shown in Figure 9 (SEI, 2021) [29]. It allows for the characterization of land use and/or soil type impacts on these processes. The Dordi catchment will be sub-divided into several sub-catchments representing different land uses/soil types aggregating the catchment area to 100% in order to observe the effect of hydrologic response in the catchment, the values of land use land cover from the individual fractional area with the catchment are summed. The surface runoff, sub-surface runoff, and baseflow are connected to the river feature, and Evapotranspiration will be lost from the system in this process.

A water balance is computed for each fractional area, j of N, assuming the constant climate over each sub-catchment. When the appropriate link is made between the catchment unit node and a groundwater node, the deep percolation within the catchment unit can be transmitted to a surface water body as base flow or directly to groundwater storage. The expression of the water balance is presented as (SEI, 2021) [30].

$$Rd_{j}\;\frac{dZ_{1,j}}{dt}=P_{e}\left(t\right)-ET_{0}\left(t\right)K_{c,j}\left(t\right)\left(\frac{5Z_{1,j}-2Z_{1,j}^{2}}{3}\right)-P_{e}\left(t\right)Z_{1,j}^{RRF}-f_{j}k_{s,j}Z_{1,j}^{2}$$

(Eq 1) $$-\left(1-f_{j}\right)k_{s,j}Z_{1,j}^{2}$$

where $Z_{1,j}$ = [1,0] is the relative storage given as a fraction of the total effective storage of the root zone, $Rd_{j}$ (mm) for land cover fraction, j; $P_{e}$ (mm) is effective precipitation, $ET_{0}\left(t\right)$ is reference evapotranspiration (mm/day), $K_{c,j}$ is the crop/plant coefficient for each fractional land cover, RRFj is the Runoff Resistance Factor of the land cover, $P_{e}\left(t\right)Z_{1,j}^{RRF}$ is the surface runoff, $f_{j}k_{s,j}Z_{1,j}^{2}$ is interflow from the first layer of land use, $f_{j}$ is partitioning coefficient relating to the land cover type, soil, and topography for the area which divides flow into horizontal $f_{j}$ and vertical $\left(1-f_{j}\right)$ & $k_{s,j}$ is the estimate of the root zone saturated conductivity (mm/time). Thus, total surface and interflow runoff, RT, from each sub-catchment at time t is given as,

(Eq 2) $$RT\left(t\right)=\sum_{j=0}^{N}A_{j}\left(P_{e}\left(t\right)Z_{1,j}^{RRF}-f_{j}k_{s,j}Z_{1,j}^{2}\right)$$

The base flow emanating from the second bucket where no return flow link is created from a catchment to a groundwater node will be calculated as below:

(Eq 3) $$S_{\max}\frac{dz_{2}}{dt}=\left(\sum_{j=1}^{N}\left(1-f_{j}\right)k_{s,j}Z_{1,j}^{2}\right)-k_{s2}Z_{2}^{2}$$

Where $S_{\max}$ is the deep percolation from the upper storage, and $k_{s2}$ is the saturated conductivity of the lower storage (mm/time).

Actual evapotranspiration (ET) is also estimated using reference ET, crop coefficient ($K_{c}$), and soil water level in the modeling unit root zone given by

(Eq 4) $$ET=ET_{0}*\;K_{c}\frac{\left(5Z_{1}-2Z_{12}\;\right)}{3}$$

$ET_{0}$ is the amount of water from the land surface which would be lost to the atmosphere when water is adequate to meet the demand for the atmospheric evaporation from the reference surface. $ET_{0}$ estimation implements the standard climatological records of humidity, sunshine, air temperature, and wind speed above an extensive surface of green grass, shading the ground, and not short of water.^44^ The Penman-Monteith method to compute $ET_{0}$ is presented as below:

(Eq 5) $$ET_{0}=\frac{0.408\Delta \left(R_{n}-G\right)+\gamma \;\frac{900}{T+273}u_{2}\left(e_{s}-e_{a}\right)}{\Delta +\gamma \left(1+0.34u_{2}\right)}$$

Where, $ET_{0}$ is the reference evapotranspiration (mm/day), $R_{n}$ is net radiation at the crop surface (MJ/m^2^day), G is soil heat flux density (MJ/m^2^day), T is mean daily air temperature at 2 m height (°C), $u_{2}$ is the wind speed at 2 m height (m/s), $e_{s}$ is the actual vapor pressure (kPa), $e_{s}-e_{a}$ is saturation vapor pressure deficit (kPa), Δ is slope vapor pressure curve (kPa/°C), and γ is the psychrometric constant (kPa/°C).

### 2.4 WEAP river nodes (SEI, 2021) [30]

In WEAP, the rivers and diversions are composed from river nodes that are connected by river reaches. Other rivers may flow in from tributaries or flow out of river (diversions). In WEAP, river nodes are categorized as follow:

**Reservoir** nodes: They represent reservoir sites on the river. Water can be directly released to demand sites or for use downstream via river reservoir node. They can be also used to simulate hydropower generation.

**Run-of-river** hydropower nodes: They represent points in the WEAP model on which run-of-river hydropower stations are located. These hydropower stations generate power on the basis varying streamflow but a constant water head in the river.

**Flow requirement** nodes: They maintain the minimum instream flow required at a point on a river or diversion in order to meet requirement of water quality, Aquatic & wildlife, navigation, recreation, downstream or other any requirements.

**Withdrawal** nodes: They represent points where any number of demand sites receive water directly from a river.

**Diversion** nodes: The function of these nodes in WEAP is to divert water from a river or other diversion into a canal or pipeline called a diversion. This diversion itself is like a river, comprised of a series.

### 2.5 WEAP algorithms for hydropower generation (SEI, 2021) [30]

#### 2.5.1 Run-of-river hydropower flows

The flow releasing out of the facility is the sum of the flow in from upstream, demand site (*DS*) and treatment plant (*TP*) return flows that come in at that point.

(Eq 6) *DownstreamOutflow*_*ROR*_*= UpstreamInflow*_*ROR*_*+ DSReturnFlow*_*DS,ROR*_*+ TPReturnFlow*_*TP,ROR*_

Hydropower generation is calculated from the amount of water flows through the turbine, based on the reservoir release or run-of-river streamflow, which is constrained by the maximum flow capacity of turbine. The amount of water flowing through the turbine is computed differently for local reservoirs, river reservoirs and run-of-river hydropower. For river reservoirs, all water released downstream is passed through the turbines, however water pumped from the reservoir to meet the direct withdrawals from reservoir is not passed through the turbines.

(Eq 7) *Release*_*H*_*= DownstreamOutflow*_*H*_

For local reservoirs, all linked demand sites are assumed to be downstream of the reservoir, therefore all reservoir releases are passed through the turbines.

(Eq 8) *Release*_*H*_*= TransLinkInflow*_*H,DS*_*+ ExtraOutflowForHydropowerRequirement*

**For run-of-river hydropower nodes**, the "release" is equal to the downstream outflow from the node.

(Eq 9) *Release*_*H*_*= DownstreamOutflow*_*H*_

The volume of water flowing through the turbines is limited by the maximum flow of turbine. Even if there is too much water, extra water is assumed to be released through spillways but that do not contribute to generate electricity.

(Eq 10) *VolumeThroughTurbine*_*H*_*= Min(Release*_*H*_*, MaxTurbineFlow*_*H*_*)*

The gigajoules (GJ) of energy produced in a month,

(Eq 11) *EnergyFullMonthGJ*_*H*_*= VolumeThroughTurbine*_*H*_*x HydroGenerationFactor*_*H*_

is a function of the mass of water (1000 kg/mˆ3) through the turbines multiplied by the head, the plant factor (fraction of time on-line), the generating efficiency, and a conversion factor (9.806 kN/m^3^ is the specific weight of water, and from joules to gigajoules). The plant factor and efficiency of turbine-generator set are entered as data

(Eq 12) *HydroGenerationFactor*_*H*_*= 1000 (kg / mˆ3) ∗ DropElevation*_*H*_*x PlantFactor*_*H*_*x PlantEfficiency*_*H*_*∗ 9.806 / (1,000,000,000 J / GJ)*

For reservoirs, head is calculated from the difference in the elevation attained at the beginning of the month and the tailwater's elevation

(Eq 13) *DropElevation*_*H*_*= BeginMonthElevation*_*H*_*- TailwaterElevation*_*H*_

**For run-of-river hydropower nodes**, the drop in elevation is entered as data

(Eq 14) *DropElevation*_*H*_*= FixedHead*_*H*_

If a demand priority for hydropower energy has been set for an individual reservoir, WEAP will calculate the supply requirement (volume of water through the turbines) necessary to generate the energy demand.

(Eq 15) *SupplyRequirement*_*H*_*= EnergyDemandFullMonthGJ*_*H*_*/ HydroGenerationFactor*_*H*_

### 2.6 Calibration and validation of Dordi River WEAP model

**Figure 10:**
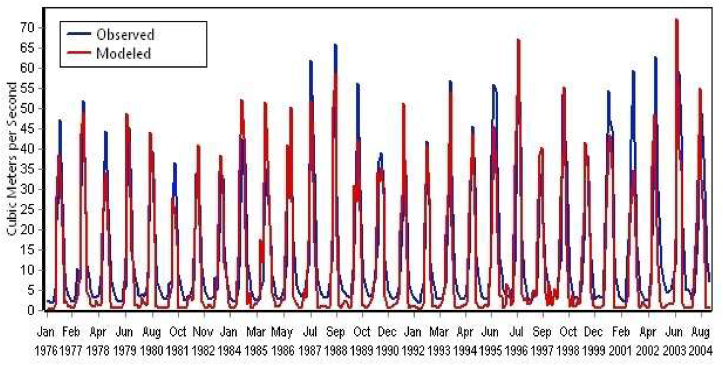
Simulated and observed streamflow WEAP results of Dordi River from 1976 to 2004.

The climatic data that includes precipitation, average temperature, relative humidity, wind speed; land use, and soil parameters, are used to simulate streamflow outputs. The simulated and observed streamflow outputs of the Dordi River from 1976 to 2004 are presented in Figure 10.

The model was calibrated to estimate the land use and soil-related parameter using the manual method. The values of land and soil parameters are selected in such a way it will give a good fit between the measured and simulated streamflow & best performance statistics results for the WEAP model [32].

### 2.7 WEAP model performance evaluation measures

The efficiency of WEAP model performance was assessed by comparing the observed streamflow versus the simulated streamflow using performance evaluation statistics – Coefficient of Determination (R2), Nash-Sutcliffe Efficiency (NSE) & Root Mean Square Error – observations Standard deviation Ratio (RSR).

Coefficient of Determination (R^2^) measures the degree of collinearity between observed and simulated values [37]. The Value of R^2^ ranges from 0 to 1. The formula for determining the value of R^2^ is given below:

$$R^{2}=\frac{\sum_{i=1}^{n}(Y_{i}^{sim}-X^{sim})\;((Y_{i}^{obs}-X^{obs})\;}{\sqrt{\sum_{i=1}^{n}{\left(Y_{i}^{sim}-X^{sim}\right)}^{2}\;\sum_{i=1}^{n}{\left(Y_{i}^{obs}-X^{obs}\right)}^{2}\;}}$$

Where, $Y_{i}^{sim}$ is the simulated streamflow, $Y_{i}^{obs}$ is the observed streamflow, $X^{sim}$ is the mean of simulated streamflow, and $X^{obs}$ is the mean of observed streamflow.

The values of $R^{2}$ that are higher than 0.5 are acceptable [33, 34]. The higher values, the lesser the error variance.

The Nash-Sutcliffe Efficiency (NSE) evaluates the hydrological model's predictive capability. The Value of NSE ranges between - ∞ and 1, where NSE = 1 shows the perfect fitness between the simulated and observed streamflow, NSE = 0 shows that the model predictions are as accurate as the mean of the observed data & NSE<0 shows that the observed mean is a better predictor than model [35]. The formula for determining the value is presented below:

$$NSE=1-\frac{\sum_{i=1}^{n}\;{\left(Y_{i}^{obs}-Y_{i}^{sim}\right)}^{2}\;}{\sum_{i=1}^{n}\;{\left(Y_{i}^{obs}-X^{obs}\right)}^{2}\;}$$

Where, $Y_{i}^{sim}$ is the simulated streamflow, $Y_{i}^{obs}$ is the observed streamflow & $X^{obs}$ is the mean of observed streamflow.

RSR is the ratio of Root Mean Square Error to Standard deviation. The formula for determining the RSR is given below:

$$RSR=\frac{\sum_{i=1}^{n}\;{\left(Y_{i}^{obs}-Y_{i}^{sim}\right)}^{2}\;}{\sum_{i=1}^{n}\;{\left(Y_{i}^{obs}-X^{sim}\right)}^{2}\;}$$

Where, $Y_{i}^{sim}$ is the simulated streamflow, $Y_{i}^{obs}$ is the observed streamflow & $X^{sim}$ is the mean of simulated streamflow.

Percent bias (PBIAS) measures the average tendency of the simulated data to be larger or smaller than their observed counterparts. The optimal value of PBIAS is 0.0, with low-magnitude values indicating accurate model simulation. Positive values indicate model underestimation bias, and negative values indicate model overestimation bias. The formula for determining the PBIAS is given below:

$$PBIAS=\frac{\sum_{i=1}^{n}\;\left(Y_{i}^{obs}-Y_{i}^{sim}\;\right)*\left(100\right)}{\sum_{i=1}^{n}\left(\;Y_{i}^{obs}\right)\;}$$

Where, $Y_{i}^{sim}$ is the simulated streamflow, $Y_{i}^{obs}$ is the observed streamflow

## 3 Result and discussion

This section assesses 1) model performance of Dordi river using performance evaluation measures - $R^{2}$, NSE & RSR 2) the impacts on streamflow and hydropower generation due to the variation in the climatic parameters. The model performance of Dordi river has shown Goodness of fit measure as **Good** and **Very Good.** Similarly, after performing the WEAP modeling of the Dordi river, it has been observed that there will be an overall increment in the streamflow of the Dordi river and hydropower generation of Super Dordi HPP under Climatic Scenario -1, 2 & 3.

### 3.1 WEAP model performance evaluation

**Figure 11:**
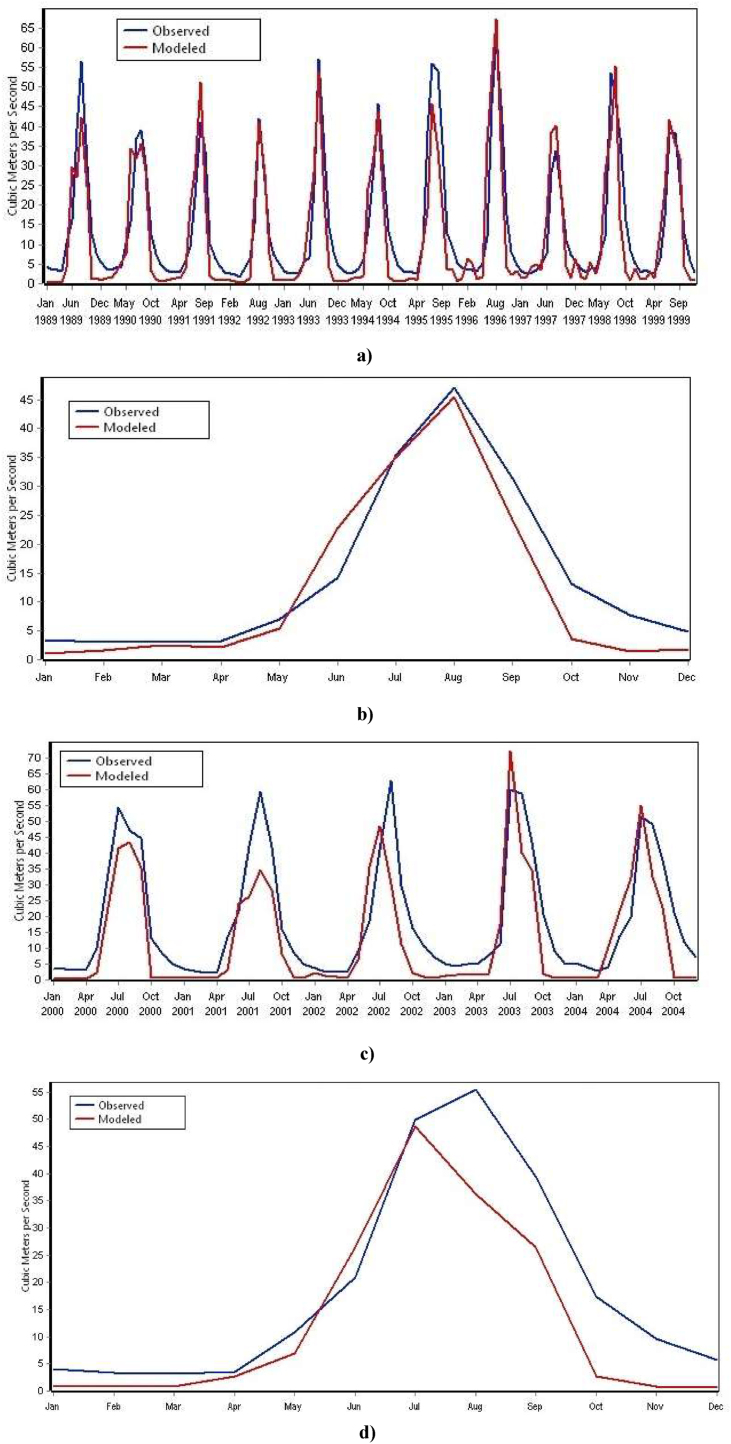
a): Observed & simulated monthly streamflow for calibration period b): Observed & simulated mean monthly streamflow for calibration period c): Observed & simulated Monthly streamflow for validation period d): Observed & simulated Mean monthly streamflow for validation period.

In this study, the monthly observed streamflow data of the Dordi River from 1989 to 1999 was used to calibrate the WEAP model, and the observed streamflow data from 2000 to 2004 was used to validate the model. Such calibrated and validated WEAP results are shown in Figure 11 a), b), c) & d).

For the monthly data, the values between

0.75 < NSE ≤1, 0 ≤ RSR ≤0.5 and PBIAS < ± 10, is rated as very good.
0.65 < NSE ≤0.75, 0.5 ≤ RSR ≤0.6 and PBIAS < ± 15, is rated as good.
0.5 < NSE ≤0.65, 0.6 ≤ RSR ≤0.7 and PBIAS < ± 25, is rated as satisfactory.
NSE ≤0.5, RSR >0.7 and PBIAS > ± 25, is rated unsatisfactory (Moriasi et al., 2007) [36].

The model performance of Dordi river was performed to simulate the mean monthly streamflow with $R^{2}$, NSE, RSR & PBIAS values of 0.91, 0.87, 0.34 & -10 respectively for the calibration period of 1989–1999. Similarly, monthly streamflow with $R^{2}$, NSE, RSR & PBIAS values of 0.81, 0.75, 0.5 & -10 respectively for the same calibration period. Thus, by the above expression, this result has indicated a very good agreement between the mean monthly observed and simulated streamflow in the Dordi river. Likewise, the result has indicated a good agreement between monthly observed and simulated streamflow.

For the validation period from 2000 to 2004, the model performance of the Dordi river was conducted to simulate the mean monthly streamflow with $R^{2}$, NSE, RSR & PBIAS values of 0.9, 0.82, 0.4 & -25 respectively. Similarly, the model performance was conducted to simulate the monthly streamflow $R^{2}$, NSE, RSR & PBIAS values of 0.78, 0.7, 0.54 & -25 respectively, for the validation period. Thus, the result has shown a very good and satisfactory agreement. The Performance Statistics of the Dordi river model for measured and simulated monthly and mean monthly streamflow are summarized and presented in Table 4.

**Table 4:** Model performance statistics summary for measured and modeled Dordi River – Monthly and mean monthly streamflow.

Statistics	Monthly	Mean Monthly
**Calibration Period**	**1989–1999**	
Coefficient of Determination (R^2^)	0.81	0.91
Nash-Sutchliffe Coefficient (NSE)	0.75	0.87
RMSE-observations Standard Deviation	0.5	0.34
Ratio (RSR)		
Percent BIAS	-10%	-10%
**Validation Period**	**2000–2004**	
Coefficient of Determination (R^2^)	0.78	0.9
Nash-Sutchliffe Coefficient (NSE)	0.7	0.82
RMSE-observations Standard Deviation	0.54	0.4
Ratio (RSR)		
Percent BIAS	-25%	-25%

The WEAP River model of this study has been also validated by comparing it with WEAP hydrological model performance evaluation of other similar studies conducted across different parts of the world. The study in the Central Rift Valley basin of Ethiopia [32] revealed the WEAP hydrological model to achieve the $R^{2}$ & NSE 0.82, 0.8; 0.91 & 0.91 for the monthly calibration and validation periods between observed and simulated streamflow, respectively. Another study in the USA [27] had developed the WEAP hydrological model to achieve the $R^{2}$ & NSE 0.92, 0.91; 0.83 & 0.78 for the monthly calibration and validation periods between observed and simulated streamflow respectively. The $R^{2}$ & NSE 0.85, 0.86; 0.89 & 0.87 were attained between observed and simulated streamflow in the Central Indus basin [37]. Therefore, these previous studies have confirmed the capability of the WEAP hydrologic model in reproducing catchment hydrology processes in a different part of the world.

### 3.2 Streamflow

**Figure 12:**
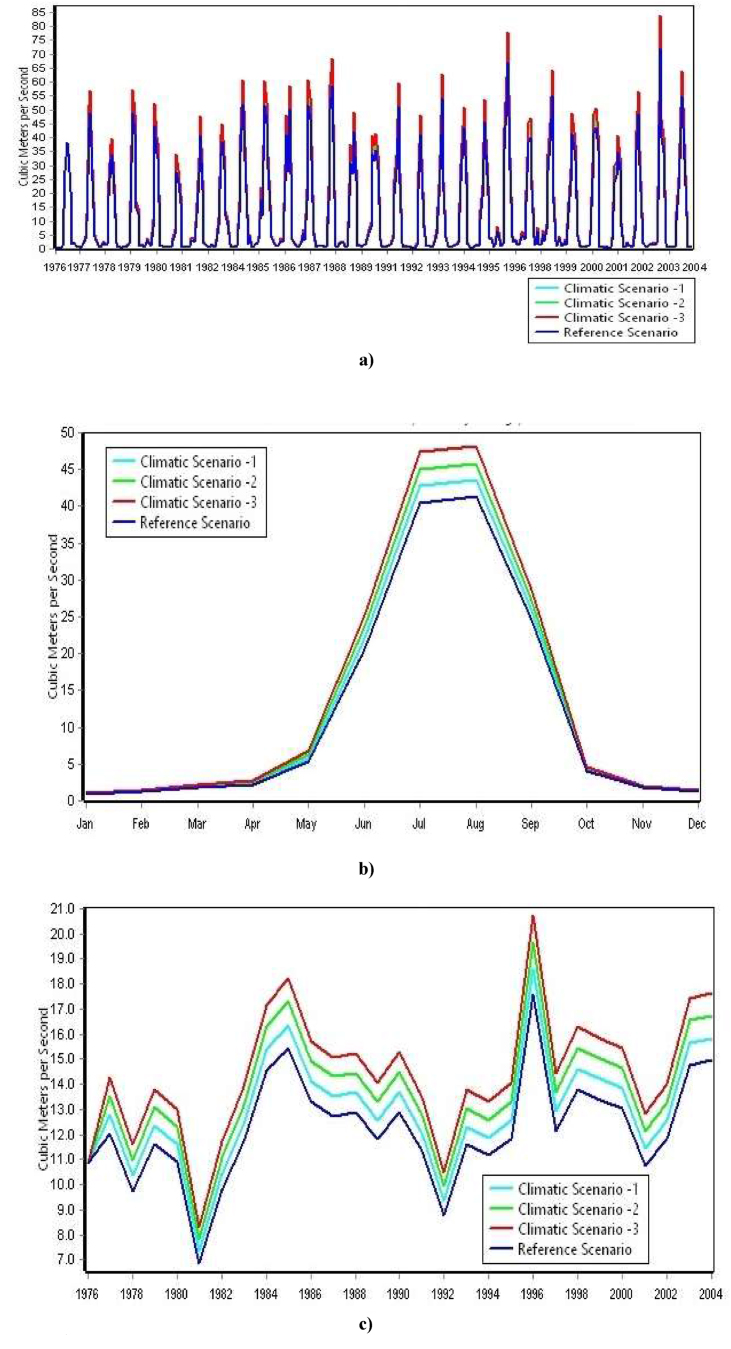
a): Results for simulated streamflow of Dordi River from 1976 to 2004 under reference & climatic scenarios b): Results for monthly average streamflow under reference & climatic scenarios c): Results for annual total streamflow from 1976 – 2004 under reference & climatic scenarios.

The streamflow of the Dordi river is observed to increase up to 15%, 1%–32% & 1%–51% over the modeling period under i) Climatic Scenario -1: when the temperature & precipitation is increased by 0.5 °C & 5 %, ii) Climatic Scenario -2: when the temperature & precipitation is increased by 1 °C & 10 % & iii) Climatic Scenario -3: when the temperature & precipitation is increased by 1.5 °C & 15 %, respectively, as compared to simulated values of streamflow under Reference Scenario which is represented by Figure 12 a) & c).

Moreover, the results of the study under these scenarios revealed a more prominent increase in the streamflow of the Dordi River during the April, May, June & July months of the season due to the increment of climatic parameters under the above mentioned Climatic Scenarios which is represented by Figure 12b shown above.

### 3.3 Hydropower generation

**Figure 13:**
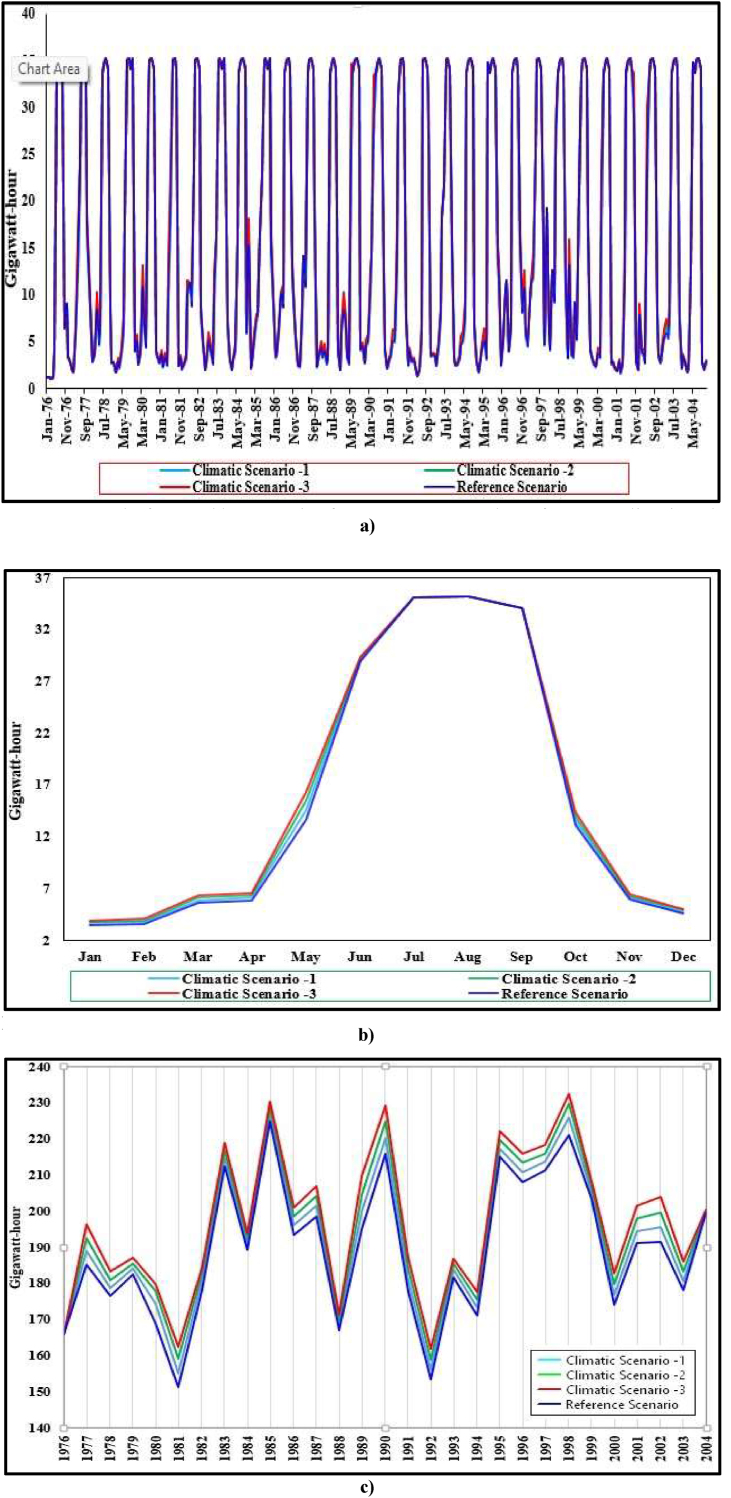
a): Results for monthly generation from 1976 to 2004 under reference & climatic under reference & climatic scenarios b): Results for monthly average generation under reference & climatic scenarios c): Results for total annual generation from 1976-2004 under ref & climatic scenarios. Note: The maximum plant discharge will be limited by turbine's maximum design flow capacity. Therefore, plant can generate power up to the maximum generation flow capacity of turbine and installed capacity of plant.

Likewise, the power generation of the plant is found to be increased over the modeling period from up to 15%, 1%–32% & 1%–51% under Climatic Scenario -1, 2 & 3, respectively, as compared to the simulated values of the hydropower generation under Reference Scenario which is represented by Figure 13 a) & c) as shown below.

After a detailed assessment of the Study's results, it has been found that there is an increment in hydropower generation of the plant during dry seasons & this increment can be mainly pronounced during April & May of the season. However, there are no impacts on the generation of power plants, mainly during June, July, August & September of the wet season when the temperature & precipitation both are increased simultaneously under Scenario -1, 2 & 3 which is represented by Figure 13 b).

This is because the streamflow at Reference Scenario during the dry seasons is low as compared to the plant's design discharge. Thus, the increment of the streamflow under the Climatic Scenario during the dry season results in the increment of the hydropower generation of plants under Climatic Scenario during dry seasons.

On the Contrary, the streamflow during the wet season at the Reference Scenario is already higher than plant design discharge in major cases. Therefore, the increment of the streamflow under the Climatic Scenario has no significant impact on hydropower generation during the wet season.

## 4 Conclusions and recommendations

### 4.1 Conclusions

In this research, the WEAP hydrological model that was calibrated & validated by historical data was implemented to model the Dordi river between 1976 to 2004. This study concludes that there are prominent impacts on the streamflow of the Dordi river and hydropower generations due to the variation of climatic parameters. Base on the study findings, following conclusions can be drawn:

1. The streamflow of the Dordi river is observed to increase up to 15%, 1%–32% & 1%–51% over the modeling period under Climatic Scenario -1, 2 & 3, respectively, as compared to the simulated values of streamflow under Reference Scenario. These increments are more prominent during the April to July months of the season.
2. The power generation of Super Dordi HPP is projected to increase up to 15%, 1%–32% & 1%–51% under climatic scenario-1, 2 & 3, respectively, as compared to baseline scenario and the increments can be mainly pronounced during April & May of the season. However, there are no impacts on the generation of power plants, mainly during June, July, August & September of wet season under Climatic Scenarios -1, 2 & 3.

This type of site-specific research will certainly assist in better analysis of the collective assessment of climate change's impact on hydropower.

### 4.2 Recommendations

1. As it can be observed from the above results, the streamflow is dynamically changing with the variation of the climatic conditions; therefore, it is necessary to analyze the varying hydrological conditions of the Dordi River with the constant provision of the monitoring system. The rainfall gauging in the climatic station & discharge measurements in the Dordi River shall be conducted from time to time for more updated and accurate data for analysis. Thus, the hydrological curve of the turbine shall be designed and selected considering the possible dynamics of streamflow in the Dordi River in the future due to the variation of climatic parameters to obtain the optimum outcome. The revision of the design discharge of the plant shall be carried out in the future in accordance with the projected discharge.
2. Similarly, the efficiency curve and power capability curve of the plant's generator shall be designed and selected considering the potential increment of generation in a hydropower plant in the future due to climatic variation.
3. Moreover, the results of this study revealed that the generation of the power plant is likely to increase due to the variation of climatic parameters during the dry season which will have significant impacts on the energy development, planning, and implementation in the context of Nepal. Thus, proper technical actions shall be taken prior to or during the development of hydropower project to enhance the power generating capability of the hydropower plant.
4. The unit commitment and scheduling of the hydropower plant shall be done in accordance with increment patterns of streamflow and hydropower generation due to the variation in climatic parameters under climatic scenarios.

## Declarations

### Author contribution statement

Raj Singh: Conceived and designed the experiments; Performed the experiments; Analyzed and interpreted the data; Contributed reagents, materials, analysis tools or data; Wrote the paper.

Nawraj Bhattarai; Shree Raj Shakya; Anita Prajapati: Contributed reagents, materials, analysis tools or data.

### Funding statement

This research did not receive any specific grant from funding agencies in the public, commercial, or not-for-profit sectors.

### Data availability statement

Data included in article/supp. material/referenced in article.

### Declaration of interest's statement

The authors declare no competing interests.
